# Postoperative hyponatremia in neonates with esophageal atresia and tracheoesophageal fistula receiving restricted hypotonic fluids

**DOI:** 10.1186/s43159-022-00197-w

**Published:** 2022-09-21

**Authors:** Shivani Dogra, Muneer A. Malik, Nitin J. Peters, Ram Samujh

**Affiliations:** Department of Pediatric Surgery, Advanced Pediatric Centre, Postgraduate Institute of Medical Education& Research (PGIMER), Chandigarh, 160012 India

**Keywords:** Hyponatremia, Syndrome of inappropriate antidiuretic hormone secretion (SIADH), Tracheoesophageal fistula

## Abstract

**Background:**

During the postoperative course following neonatal surgery, several stimuli like respiratory distress, pain, and stress cause the release of the antidiuretic hormone which can induce hyponatremia. This hyponatremia due to syndrome of inappropriate antidiuretic hormone secretion (SIADH) in neonates can lead to neurologic impairment and in severe cases can cause significant morbidity and mortality. Lung involvement in neonates undergoing TEF makes this subset of patients vulnerable to this entity because most of them are sick and require ventilation in the postoperative period. The incidence of postoperative hyponatremia following neonatal surgery has not been studied vastly. To the best of our knowledge, this is the first prospective study that has analyzed the incidence of postoperative hyponatremia in this vulnerable population.

**Methods:**

Prospective observational study to assess the incidence of postoperative hyponatremia in neonates with esophageal atresia and tracheoesophageal fistula (EA and TEF) receiving restricted hypotonic fluids. As per the unit policy N/4 5% D is given in the postoperative period. Most neonatal units follow a protocol in which fluid is hiked daily to reach 150 ml/kg/day in 5–7 days. However, in our neonatal surgical unit a protocol to restrict the maintenance fluid at 100 ml/kg/day irrespective of day of life is followed.

**Results:**

Out of a total of 90 neonates (270 sodium measurements), we identified 16 with hyponatremia (11%). Most of the neonates had mild hyponatremia(130–135 meq/l). The incidence of moderate and severe hyponatremia was low.

**Conclusion:**

Postoperative restriction of fluids especially in neonates who are at a high risk for developing SIADH can lead to decreased incidence of severe hyponatremia.

## Background

Esophageal atresia and tracheoesophageal fistula (EA and TEF) is one of the most common congenital anomalies encountered in pediatric surgery with an incidence of approximately 1 in 3000 births [1]. An improvement in survival has been reported in the last decade due to advances in neonatal intensive care. However, the management of fluid-electrolyte balance and ventilation still poses a problem in the postoperative period. Hyponatremia in particular is a common electrolyte disturbance seen in these sick neonates. There are several stimuli for the release of antidiuretic hormone in the postoperative period, which controls renal water handling, including pain, stress, and hypovolemia. The resultant syndrome of inappropriate antidiuretic hormone secretion (SIADH) leads to an increased incidence of hyponatremia in this population. Nearly all the neonates with TEF require elective ventilation making them more vulnerable to the risk of developing SIADH and hence, hyponatremia. The main concern with hyponatremia in the neonatal population is the consequent adverse neurological outcomes [2].

Calculation of volume of maintenance fluids has been according to Holliday and Segar’s guidelines for postoperative pediatric patients [3]. However, neonatal protocols for fluid administration dictate a much higher fluid administration in neonates [4]. This excess fluid may lead to circulatory overload and difficult weaning from the ventilator. We in our unit follow a protocol of giving restricted fluids to TEF patients in the postoperative period assuming that this practice is beneficial in neonates at risk of developing SIADH.

Hypotonic solutions had widespread use for decades until the danger of induced hyponatremia was demonstrated in clinical practice and a paradigm shift took place towards the use of isotonic fluid in pediatric patients [5]. Various studies have highlighted the risk of developing hyponatremia in children who are receiving hypotonic fluid in the postoperative period [6, 7]. Now routine use of hypotonic intravenous fluid during neonatal surgery has also been questioned as it is likely to reduce plasma sodium [8]. The American Academy of Pediatrics has recommended the use of isotonic solutions with appropriate potassium chloride and dextrose in patients 28 days to 18 years of age to decrease the risk of hyponatremia [9]. However, in sick neonates evidence-based consensus guidelines regarding the type of fluids are still lacking.

The incidence of postoperative hyponatremia in the neonatal population has not been studied vastly. The guidelines regarding the type and volume of fluids in this vulnerable population have not been clearly outlined. In our unit, we follow protocol to provide a restricted fluid to the neonates with EA and TEF in the postoperative period with an assumption that this policy leads to less interstitial edema and less SIADH thus decreasing the incidence of severe hyponatremia.

## Methods

This is a prospective single centre observational study undertaken with ethics committee approval (NK/7259/STUDY/126). It was conducted in the neonatal surgical intensive care unit of a tertiary care referral hospital in North India from August 2020 to May 2021. The inclusion criteria were term and late preterm neonates (> 34^0/7^weeks) ventilated in the postoperative period post TEF surgery. Neonates with pure esophageal atresia were excluded from the study. Other exclusion criteria were neonates with complex cardiac disease, creatinine more than 1.5 mg/dl in the preoperative period, or inotropes more than 2 in the postoperative period.

After optimum fluid, electrolyte and respiratory stabilization all neonates with EA+TEF underwent surgical repair. In the postoperative period all neonates were electively ventilated for at least 48–72 h and then extubated as per the clinical status assessed by the treating team. As per the unit policy, N/4 5% D was given in the postoperative period as restrictive fluid therapy. Most neonatal units follow a protocol in which fluid is hiked daily to reach 150 ml/kg/day in 5–7 days [6]. However, in our neonatal surgical unit, a protocol to restrict the maintenance fluid at 100 ml/kg/day irrespective of the day of life is followed. Further titration of fluid if required, was done on the basis of the clinical hydration status of the neonate. Trophic feeds were started 24 to 48 h postoperatively as per the clinical status of the neonates and then gradually increased. The amount of feed tolerated was deducted from the total maintenance fluid with total quantity being not more than 100 ml/kg. The primary outcome of the study was to estimate the incidence of hyponatremia in neonates undergoing surgery for esophageal atresia and TEF receiving restricted hypotonic maintenance fluids. Serum sodium concentrations were measured as part of our routine laboratory practice in all neonates in the postoperative period using an ABL 8000 basic analyzer. This sampling is done every morning as per the unit protocol in all the sick neonates. If sodium was repeated for any reason within 24 h it was not considered for analysis. Hyponatremia was defined as a sodium level less than 135 meq/L. It was further sub-classified as mild, moderate, and severe based on values (mild 130–135 meq/L, moderate < 130–125 meq/L and severe < 125 meq/L). Baseline variables including birth weight, gender, gestation, were recorded in a proforma. Episodes of hyponatremia were managed as per the standard protocol. Charts were reviewed daily and various other parameters such as daily fluid intake, urine output, inotropes were recorded. The outcome of the baby was also noted. Clinical characteristics of neonates with or without hyponatremia were analyzed.

### Statistical analysis

In this prospective observational study, we studied the incidence of postoperative hyponatremia in neonates undergoing surgery for tracheoesophageal fistula and analyzed factors associated with the development of this dyselectrolytemia. Statistical analysis was carried out using Statistical Package for Social Sciences (SPSS). Data were summarized as means with SDs or frequencies with percentages for continuous and categorical variables respectively. A univariate analysis was performed—quantitative variables with central tendency were described using means and standard deviations. For variables with non-normal distribution, medians and interquartile ranges were used. Likewise, qualitative variables were reported using percentages Parametric data were analyzed using *t* test and non-parametric data was analyzed using Wilcoxon-Mann-Whitney *U* test. The normalcy of quantitative data was checked using Kolmogorov–Smirnov test. All tests were 2 tailed with 95% confidence interval and level of significance 5% (*p* < 0.05). Data is presented in tabulation/graphical forms.

## Results

One hundred twenty-six neonates with esophageal atresia were admitted to NSICU between August 2020 to May 2021. After the application of exclusion criteria (pure esophageal atresia = 14, complex cardiac disease = 5, > 2 inotropes = 6, S. creatinine > 1.5 = 4, refusal to consent = 7), 90 neonates were found eligible for inclusion in the study. Nearly all the neonates who were shifted to the surgical ICU were outborn. The initial part of the resuscitation and management was done in emergency where they stayed for a variable period of time during the preoperative phase. This study was conducted during the COVID-19 pandemic which delayed the surgery as covid appropriate investigations were carried out. The median age at which surgery was done was 4(2–7) days. The majority of the neonates were term or late preterms and a mean gestational age of 36.6 ± 1.5 weeks was seen (Table 1). The mean serum sodium levels in preoperative periods were 136 ± 3.6 meq/L.

**Table 1:** Baseline characteristics of the enrolled neonates

Variables	Value
Gestation (week), mean ± SD	36.6 ± 1.5
Birth weight (kg)mean ± SD	2.39 ± 0.3
Gender, females (*n*%)	38%
Median age at surgery in days (IQR)	4(2–7)
Preoperative sodium (meq/L)mean ± SD	136 ± 3.5
Associated anomalies (*n*%)	35%

Postoperative characteristics of the studied neonates have been depicted in Table 2. Out of a total of 90 neonates (270 sodium measurements), we identified 21 patients with hyponatremia (23%). Most of the neonates had mild hyponatremia (130–135 meq/l). The incidences of moderate and severe hyponatremia was 3%. The average fluid intake on days 1, 2, and 3 postoperatively was 105 ml/kg/day, 106 ml/kg/day, and 103 ml/kg/day. The average fluid intake on days 1, 2, and 3 postoperatively was 105 ml/kg/day, 106 ml/kg/day, and 103 ml/kg/day (Table 3) which was much less than the expected fluid requirement for that age. Considering the fact that these neonates were operated on at a median age of 4 days so expected fluid requirement as per the routine neonatal protocols would be 120 ml/kg/day, 140 ml/kg/day, and 150 ml/kg/day on day 1, 2, and 3 postoperatively respectively. Lactate was high (3.1 ± 0.8 mmol/l) in the immediate postoperative period and gradually decreased (1.9 ± 0.7 mmol/L) over the next 48 h. (Normal lactate values in neonates being less than 2 mmol/L).

**Table 2:** Postoperative characteristics of the neonates

Variable	Value
Hyponatremia	21/90(23%)
‘Inotropes (postop) *n*%	73/90(81%)
Ventilated in postoperative period *n*%	89/90(98.8%)
Received Lasix in postoperative period.	26/90 (28%)
Ventilation days. Median(IQR)	8(3–25)
Sepsis (*n*%)	36/90(40%)
Anastomotic leak (*n*%)	23/90 (26%)
Mortality (*n*%)	24/90(26%)

**Table 3:** Postoperative fluid and electrolyte data of the neonates

	Day 1 postop		Day 2 postop		Day 3 Postop	
**Fluid ml/kg/day (mean ± SD)**	Obs^a^	Exp^b^	obs^a^	Exp^b^	Obs^a^	Exp^b^
	101 ± 8	120	105 ±8	140	104 ± 7.9	150
**Urine output ml/kg/h (mean ± SD)**	1.4 ± 0.3		2.5 ± 0.4		2.8 ± 0.3	
**Serum sodium meq/L (mean ± SD)**	137.7 ± 4.1		139.4 ± 6		138.3 ± 4	
**Hyponatremia** ***n*%**	13/90(14%)		9/88(10%)		8/85(9.4%)	
Mild	5/90		4/88		5/85	
Moderate	7/90		5/88		3/85	
Severe	1/90		0/88		0/85	
**Serum osmolality mosm/kg (mean ± SD)**	277 ± 5		278 ± 5.5		276 ± 5.8	
**S. Lactate mmol/l(mean ± SD)**	3.1 ± 0.8		2.3 ± 0.9		1.9 ± 0.7	

^a^*Obs* observed

^b^ *Exp* expected

Urine output was slightly less in the immediate postoperative period; however, it improved after 24 h.

Univariate analysis of variables between neonates with and without hyponatremia was done (Table 4). Mortality in the neonates with moderate and severe hyponatremia was significantly higher than those without any hyponatremia records. The mortality was 71% in neonates with hyponatremia in contrast to 24% in neonates without hyponatremia. The rate of culture-positive sepsis was also higher in the neonates who had hyponatremia (57% vs 34.7%).

**Table 4:** Comparison of outcomes among neonates with and without hyponatremia

	Neonates with hyponatremia (***n*** = 21)	Neonates without hyponatremia (***n*** = 69)	*P* value
Ventilation days. ^a^	8.2 ± 4.6	5.5 ± 2.5	0.0008
Inotropes (*n*%)	80%	75%	0.63
Urine output (Avg for 3 days) ml/kg/h ^a^	1.6 ± 0.6	2 ± 0.6	0.008
Length of hospital stay ^a^	11.7 ± 8.7	11.2 ± 5.6	0.75
Avg fluid received ^a^	102.7 ± 9.9	104 ± 7.9	0.53
Seizures (*n*%)	2(9.5%)	1(1.4%)	0.07
Sepsis (*n*%)	12(57%)	24(34.7%)	0.06
Mortality (*n*%)	15(71%)	17(24%)	0.0001

^a^Mean ± SD

## Discussion

Hyponatremia is among the most common electrolyte abnormalities encountered in the perioperative period [10]. Hyponatremic encephalopathy is a serious complication that can result permanent neurological injury or even death [11]. The threshold for seizure activity and abnormal neurological examination secondary to hyponatremia is variable; however, symptoms are more likely to occur at sodium levels less than 125 mEq/L (< 125 mmol/L) [12]. Sodium levels between 130 and 135 described as mild hyponatremia in our study may not have any clinical significance especially in neonatal population. A varied incidence of hyponatremia has been described in pediatric patients undergoing surgical procedure depending on the definition of hyponatremia, patient selection, and the setting of postoperative care [13, 14]. The cause of postoperative hyponatremia is predominantly by a combination of nonosmotic stumuli for ADH release including volume depletion, pain, nausea, stress, narcotics, and the administration of hypotonic fluids [15]. Incidence of hyponatremia is even higher in neonatal population. Pooled data from literature in neonatal intensive cares shows a very high mean incidence of hyponatremia (40%) [13]. The population of neonates undergoing TEF repair may be especially vulnerable to this entity because most of them are sick and ventilated in postoperative period. The basis for this patient selection is based on our assumption that in thoracic surgeries restrictive fluid regimes can lead to less interstitial edema which can facilitate early extubation. Also, this group of neonates is at a higher risk of developing SIADH.

Hyponatremia was more common in our study in immediate 24-h postoperative period as compared to later because of possible contribution of SIADH. Another reason could be dilutional hyponatremia secondary to extra fluid received by these neonates during surgery. Urine output was significantly less in neonates who developed hyponatremia despite receiving comparable average fluid during the postoperative period. SIADH can be the most plausible explanation for this finding.

There has been a paradigm shift in the fluid management of sick children over the last decade. Isotonic fluids are now used routinely for intraoperative maintenance in children [16]. Karen Choong et al evaluated the risk of hyponatremia following administration of isotonic compared to a hypotonic fluid for 48 h to postoperative pediatric patients. Hypotonic fluids significantly increased the risk of hyponatremia, compared with isotonic fluids (40.8% vs 22.7%; relative risk 1.82) [17]. There are several other trials and guidelines recommending isotonic fluids in sick pediatric population. However, there are a very few trials comparing isotonic versus hypotonic fluids in neonates. A consensus has not yet been reached due to concerns regarding the ability of the neonate to handle salt solutions. The clinical practice guidelines of the American Academy of Pediatrics have also excluded the neonates from the consensus to use of isotonic maintenance fluids in neonates due to lack of data. We also continue to use hypotonic fluids in our unit but with volume restriction.

It may not be sufficient to use isotonic fluids alone to decrease the incidence of hyponatremia in critically ill children. Even with isotonic maintenance, more than 20% of children were diagnosed with hyponatremia [18]. Fluid restriction along with the use of isotonic fluids can decrease the risk of hyponatremia in sick children. As per the NICE guidelines, if there is a risk of water retention associated with non-osmotic ADH secretion, consider restricting fluids to 50–80% of the routine maintenance needs [19]. As free water excretion is altered for all children in the postoperative period, reduction in the volume of maintenance fluid therapy to half the previously recommended volume has been suggested by a few authors [12]. Probably, the incidence of hyponatremia in our unit where we are using hypotonic fluids was comparable to other units using isotonic fluids because of our policy of restricting the fluids. As per most neonatal protocols, fluids are started at 60 ml/kg in term neonates and 80 ml/kg in preterm neonates. This maintenance is hiked by 15–20 ml/kg to reach a fluid infused at 150 ml/kg/day [6]. But we have a protocol to keep the intravenous fluids restricted even with the advancement of age unless clinically indicated. Frusemide has also been used in a large number of neonates in the postoperative period to maintain a restrictive intravascular volume.

Various outcomes were compared among neonates with and without hyponatremia. Hyponatremia in the postoperative period is an important risk factor for mortality which was evident in our study too [20]. Sepsis was also significantly more in neonates with hyponatremia. Association between sepsis and hyponatremia has been vastly described in Literature [21].

The incidence of seizures was more in children with hyponatremia, even though the neurological status of these babies may not be adequately assessable due to concomitant sedation. There may be a concern about impaired organ perfusion with a restrictive fluid regime. However, we did not observe worsening renal functions and urine output also remained adequate. Lactate level was also not affected because of this practice of fluid restriction.

The major limitation of this study is the absence of a control group receiving non-restricted fluid, however, considering our unit practice it would have been unethical to give extra fluids to the neonates whom we consider to be at an increased risk for developing SIADH. We do not routinely do urine osmolality which could have strengthened our hypothesis.

We assume that the incidence of severe postoperative hyponatremia in our unit is less than documented worldwide due to our policy of fluid restriction and use of Lasix however, the incidence can further be reduced if we use isotonic fluids as per the current guidelines in sick pediatric patients. Further studies are required to be conducted in the neonatal population to evaluate the benefits of using isotonic fluids in the perioperative period.

## Data Availability

All data and material are available.
